# Chromosomal coharboring of *bla*_*IMP-60*_ and *mcr-9* in *Enterobacter asburiae* isolated from a Japanese woman with empyema: a case report

**DOI:** 10.1186/s12879-022-07730-7

**Published:** 2022-09-30

**Authors:** Yusuke Miyazato, Noriko Iwamoto, Masaru Usui, Toyotaka Sato, Tohru Miyoshi-Akiyama, Maki Nagashima, Kazuhisa Mezaki, Kayoko Hayakawa, Norio Ohmagari

**Affiliations:** 1 Disease Control and Prevention Center, National Center for Global Health and Medicine, Tokyo, Japan; 2 Laboratory of Food Microbiology and Food Safety, Department of Health and Environmental Sciences, School of Veterinary Medicine, Rakuno Gakuen University, Hokkaido, Japan; 3 Laboratory of Veterinary Hygiene, Faculty of Veterinary Medicine, Hokkaido University, Sapporo, Japan; 4 Graduate School of Infectious Diseases, Hokkaido University, Sapporo, Japan; 5 Pathogenic Microbe Laboratory, National Center for Global Health and Medicine, Tokyo, Japan; 6 Department of Clinical Laboratory, National Center for Global Health and Medicine, Tokyo, Japan

**Keywords:** *mcr-9*, *Bla*_IMP_, Chromosome, Heteroresistance, Case report

## Abstract

**Background:**

Polymyxin E (colistin) is a last-resort antibiotic to treat infections caused by carbapenemase-producing Enterobacteriaceae (CPE). However, reports of CPEs resistant to colistin have been increasing, and the *mcr* genes are emerging as resistance mechanisms. Among them, plasmid-mediate *mcr-9* is known to be associated with colistin resistance, whereas reports on chromosomal *mcr-9* and its association with colistin resistance in humans are few.

**Case presentation:**

We identified *Enterobacter asburiae* harboring *mcr-9* and *bla*_*IMP-60*_ in the pleural fluid of a patient with empyema. The long-read sequencing technique revealed that these genes were located on its chromosome. Despite the lack of exposure to colistin, the organism showed microcolonies in the inhibition circle in the E-test and disk diffusion test. Antibiotic susceptibility testing by broth microdilution confirmed its resistance to colistin.

**Conclusion:**

Our case report showed that *mcr-9* can be present not only on plasmids but also on the chromosome in *E. asburiae,* and that the presence of *mcr-9* on its chromosome may influence its susceptibility to colistin.

**Supplementary Information:**

The online version contains supplementary material available at 10.1186/s12879-022-07730-7.

## Background

Carbapenemase-producing Enterobacteriaceae (CPE) are a threat for healthcare providers because they can be resistant to many antimicrobial agents including carbapenems, and there are few reliable antimicrobial treatment options [1]. Reports of colistin-resistant CPE have improved our understanding of the mechanisms of resistance, including the acquisition of mobile colistin-resistance (*mcr*) genes (*mcr-1* to *mcr-10*) [2–4]. The *mcr* genes, especially *mcr-9,* are frequently found in *Enterobacter cloacae* complex strains, and several of these strains co-harbor metallo-beta-lactamase genes, such as *bla*_*NDM-1*_ and *bla*_*KPC-2*_ [5]. Plasmid-borne *mcr* genes conferring resistance to colistin pose a serious threat to international public health [3]; in particular, IncHI2 plasmids bearing *mcr-9* play a central role in the global spread of resistant strains [6]. While many reports of plasmid-borne *mcr* are available, there are only a few case reports on the detailed clinical course of infection and antibiotic susceptibility testing of Enterobacteriaceae carrying *mcr* and metallo-beta-lactamase genes on their chromosomes. Here, we report the case of a patient with empyema who carried *E. asburiae* harboring *bla*_*IMP-60*_ and *mcr-9* on its chromosome.

## Case presentation

The patient was a 68-year-old previously healthy woman who had suffered from a severe sore throat for a week and was admitted after being diagnosed with septic shock, acute epiglottitis, descending mediastinitis, and bilateral pleural empyema due to *Streptococcus constellatus* and *Bacteroides thetaiotaomicron*. Although the patient received an intensive care with treatment by percutaneous drainage and intravenous piperacillin/tazobactam, she experienced a relapse of empyema due to *E. asburiae* resistant to meropenem and colistin (Table 1). Multiple resistant microcolonies were present within the zone of clearing by the E-test and disc diffusion method (Additional file 1 and 2: Fig. S1, S2). We suspected the strain was heteroresitant to colistin at first, but antibiotic susceptibility testing using broth microdilution (BMD) confirmed colistin resistance (Table 2).

**Table 1:** MICs of different antibiotics (except colistin) for *E. asburiae* strain performed using the Microscan WalkAway system

Antibiotics	MIC (μg/ml)
Ampicillin	≥ 32
Ampicillin/sulbactam	≥ 32
Piperacillin/tazobactam	≥ 256
Cefazolin	≥ 32
Cefpodoxime proxetil	≥ 16
Cefmetazole	≥ 64
Ceftriaxone	≥ 16
Ceftazidime	≥ 32
Cefepime	≥ 32
Latamoxef	≥ 16
Cefpodoxime/clavulanate	≥ 4
Aztreonam	≥ 16
Meropenem	8
Amikacin	≤ 8
Gentamicin	≤ 1
Tigecycline	2
Nalidixic acid	≥ 64
Levofloxacin	2
Fosfomycin	≥ 64
Trimethoprim/sulfamethoxazole	≤ 2

*MIC* minimum inhibitory concentration

**Table 2:** Colistin susceptibility of *E. asburiae*

Methods for colistin susceptibility	Colistin MIC (μg/ml)
	*Enterobacter asburiae*
Disc diffusion method (mm)	No inhibition zone*
E-test	> 256*
Broth microdilution (BMD)	> 1024

*MIC* minimum inhibitory concentration

*Multiple micro-colonies were detected within the inhibition circle (Additional file 1 and 2: Fig. S1, S2)

**Fig. 1:**
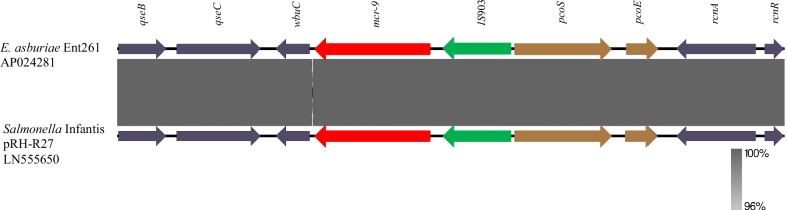
*mcr-9*-surrounding regions on the chromosome of *E. asburiae* and in a plasmid of *Salmonella Infantis*. Colored arrows represent open reading frames, with brown, green, and red arrows representing heavy metal resistance genes, mobile genetic elements, and *mcr-9*, respectively. The remaining genes are shown in gray

A detailed next-generation sequencing study was carried out to examine the mechanism of drug resistance to colistin in the *E. asburiae* strain. The long-read sequencing technique revealed that in this case, *E. asburiae* co-harbored *mcr-9* and *bla*_*IMP-60*_ on its chromosome (Fig. 1). By applying error correction twice using Pilon on short reads, an improvement in the overall sequence and length was achieved. Specifically, we performed the following analyses: (a) long-read sequencing reads were demultiplexed by Porechop v0.2.4 (https://github.com/rrwick/Porechop), and the reads were adaptor-trimmed and quality-filtered by Nanofilt (Q score, 9; minimum length, 1000 bp), (b) chimeric reads were removed using yacrd v0.6.0 (https://github.com/natir/yacrd), (c) the reads of errors were corrected by short-read sequencing reads with LoRDEC v0.8 software [7] with default parameters, (d) de novo assembly was performed by Flye v2.6 [8] (with default parameters using corrected long-read sequencing reads), (e) assembled contigs of errors were corrected by short-read sequencing reads with Pilon v1.23 [9] twice with default parameters, (f) genome and plasmid sequences were annotated using DFAST (https://dfast.nig.ac.jp), and (g) antimicrobial resistance genes were detected using ResFinder v4.1 with default parameters on the CGE server (http://www.genomicepidemiology.org). Subsequent long-read sequence analysis of *E. asburiae* using MinION revealed a circular chromosome (accession no. AP024281), which contained *bla*_*IMP60*_ and *mcr-9*. There were also resistance genes on the chromosome, such as *aac(6')-llc*, *bla*_ACT-6_, and *sul1_2*. *E. asburiae*, in this case, had only one plasmid (AP024282), but there were no resistance genes on it. A linear comparison of the *mcr-9*-surrounding regions of the chromosome of *E. asburiae* (AP024281) and a plasmid of *Salmonella* Infantis pRH-R27 was performed by BLAST and visualized using Easyfig v2.2.2 (https://mjsull.github.io/Easyfig/) (LN555650) (Fig. 1). The core structure of the reported *mcr-9* cassette, *rcnR-rcnA-pcoE-IS*903*-mcr-9-wbuC* [6], was also observed in the chromosome of *E. asburiae*.

The patient underwent repeated percutaneous drainage and intravaenous antimicrobial therapy with piperacillin/tazobactam, gentamicin and levofloxacin for two months, then we switched oral therapy with trimethoprim/sulfamethoxazole and levofloxacin until resolution of the empyema in imaging studies. After being discharged on day 168, she is now living on her own after the rehabilitation therapy and has had no recurrences.

## Discussion and conclusions

The novelty of *E. asburiae* in this case is the demonstration of colistin resistance with *mcr-9* on the chromosome. Most *mcr-9* genes are located on plasmids; a previous report stated that only 7.8% (6/78) of identified *mcr-9* genes are located on chromosomes of Enterobacteriaceae strains from human, animal, food, and the environment [6]. The *mcr* gene is believed to be mobile in various genetic elements and can be integrated into chromosomes, making colistin resistance prevalent in a large number of Enterobacteriaceae [1]. To our knowledge, this is the first report of colistin resistance in strains expressing chromosomal *mcr-9* and *bla*_*IMP-60*_. Reports of *mcr-9* on human chromosomes are rare; we summarize three cases in Additional file 3: Table S1 [10–12]. Whether there are differences in characteristics, such as susceptibility to colistin, between Enterobacteriaceae carrying *mcr-9* on plasmids and on their chromosomes is unclear.

Surprisingly, the patient in this case was infected with an *E. asburiae* strain resistant to colistin even though she had not been exposed to colistin. In our opinion, *mcr-9* and colistin resistance have not been adequately investigated. In the current case, BMD showed colistin resistance, and multiple micro-colonies were also present within the zone of inhibition circle in both E test and disk diffusion method. Napier et al*.* reported colistin susceptibility of *E. cloacae* determined using the E test; they showed that small colonies were found in the zone of inhibition [13], as in this case, which they described as “heteroresistant”. Although colistin susceptibility testing for CPE is difficult, BMD is the most reliable test [14, 15] and we judged the strain was resistant to colistin. Strains that are heteroresistant to colistin are considered to become resistant to the drug after treatment with colistin and regain sensitivity in the absence of colistin exposure. Furthermore, Kieffer et al*.* reported that the expression of cloned *mcr-9* in Enterobacteriaceae does not significantly affect colistin susceptibility and that exposure of bacteria to colistin, in addition to the introduction of *mcr-9*, may induce *mcr-9* expression and lead to colistin resistance [16]. Three previous reports showed that Enterobacteriaceae isolated from humans with *mcr-9* on the chromosome were colistin-sensitive (Additional file 3: Table S1) [10–12], whereas *E. asburiae* in the current case was resistant to colistin despite the lack of exposure to the drug. Further studies are needed to determine whether the presence of *mcr-9* on the chromosome influences clinical colistin susceptibility. Moreover, It is possible that *mcr-9* and *bla*_*IMP-*_60 gene were transferred from the *B. thetaiotaomicron* detected from the patient, although it was susceptible to carbapenem and ampicillin/sulbactam. We could not conduct a further investigation because we first initiated treatment with piperacillin/tazobactam and after the empyema recurred, microbiology testing results could not detect *B. thetaiotaomicron*. This is a limitation of this study.

In conclusion, we identified a colistin-resistant strain of *E. asburiae* that simultaneously possesses *mcr-9* and *bla*_*IMP-60*_ genes on its chromosome. The presence of *mcr-9* on the chromosome may be associated with colistin resistance.

## Supplementary Information

**Additional file 1: Supplementary Figure 1.** Image of Enterobacter asburiae isolate colR/S plated on Mueller-Hinton agar with a colistin E test strip (left) and the disc diffusion method (right). Resistant colonies were present within the zone of clearing.

**Additional file 2: Supplementary Figure 2.** Magnified image of E test's micro-colonies. Red arrows indicate resistant colonies.

**Additional file 3: Supplementary Table 1.** Case Reports of Enterobacteriaceae carrying chromosomally located mcr-9 isolated from humans.

## Data Availability

The BioProject no. of this study is PRJDB10973. Draft genome sequences were deposited at the DNA Data Bank of Japan (https://www.ddbj.nig.ac.jp/ddbj/index-e.html) and with GenBank accession numbers AP024281.1 and AP024282.1.

## Abbreviations

CPE: Carbapenemase-producing Enterobacteriaceae
*mcr*: Mobile colistin-resistance
BMD: Broth microdilution
