# Supplementary material for: Chromosomal coharboring of blaIMP-60 and mcr-9 in Enterobacter asburiae isolated from a Japanese woman with empyema: a case report
Source: BMC Infect Dis. 2022 Sep 30;22:762. doi: 10.1186/s12879-022-07730-7 (PMC9523918; doi:10.1186/s12879-022-07730-7)
Supplement: Supplementary file 1 — Additional file 1: Supplementary Figure 1. Image of Enterobacter asburiae isolate colR/S plated on Mueller-Hinton agar with a colistin E test strip (left) and the disc diffusion method (right). Resistant colonies were present within the zone of clearing. [file 12879_2022_7730_MOESM1_ESM.pdf]

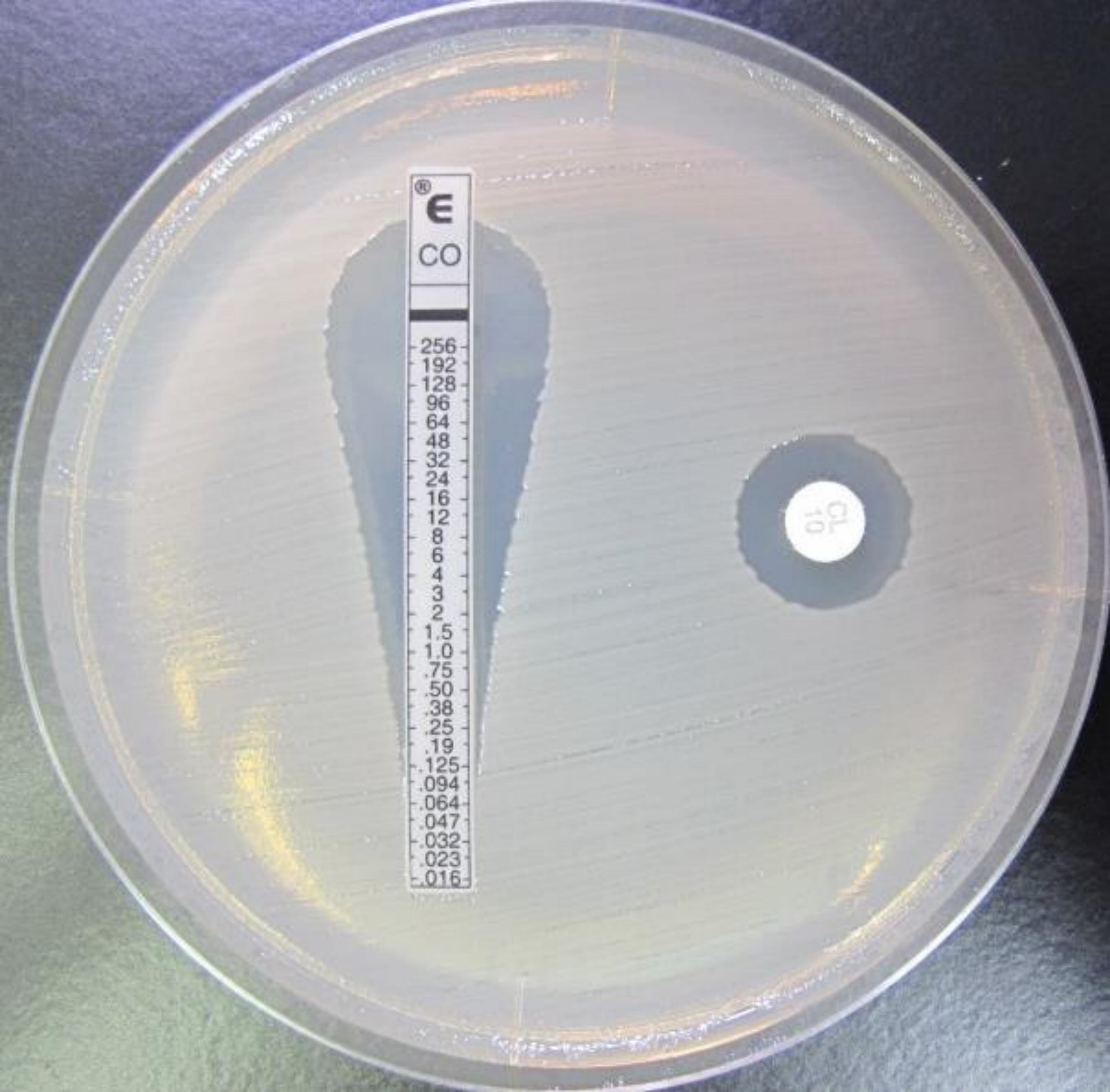

®
E
CO
256
192
128
96
64
48
32
24
16
12
8
6
4
3
2
1.5
1.0
.75
.50
.38
.25
.19
.125
.094
.064
.047
.032
.023
.016
CL
10
