# Supplementary figures and images for: Chromosomal coharboring of blaIMP-60 and mcr-9 in Enterobacter asburiae isolated from a Japanese woman with empyema: a case report
Source: BMC Infect Dis. 2022 Sep 30;22:762. doi: 10.1186/s12879-022-07730-7 (PMC9523918; doi:10.1186/s12879-022-07730-7)

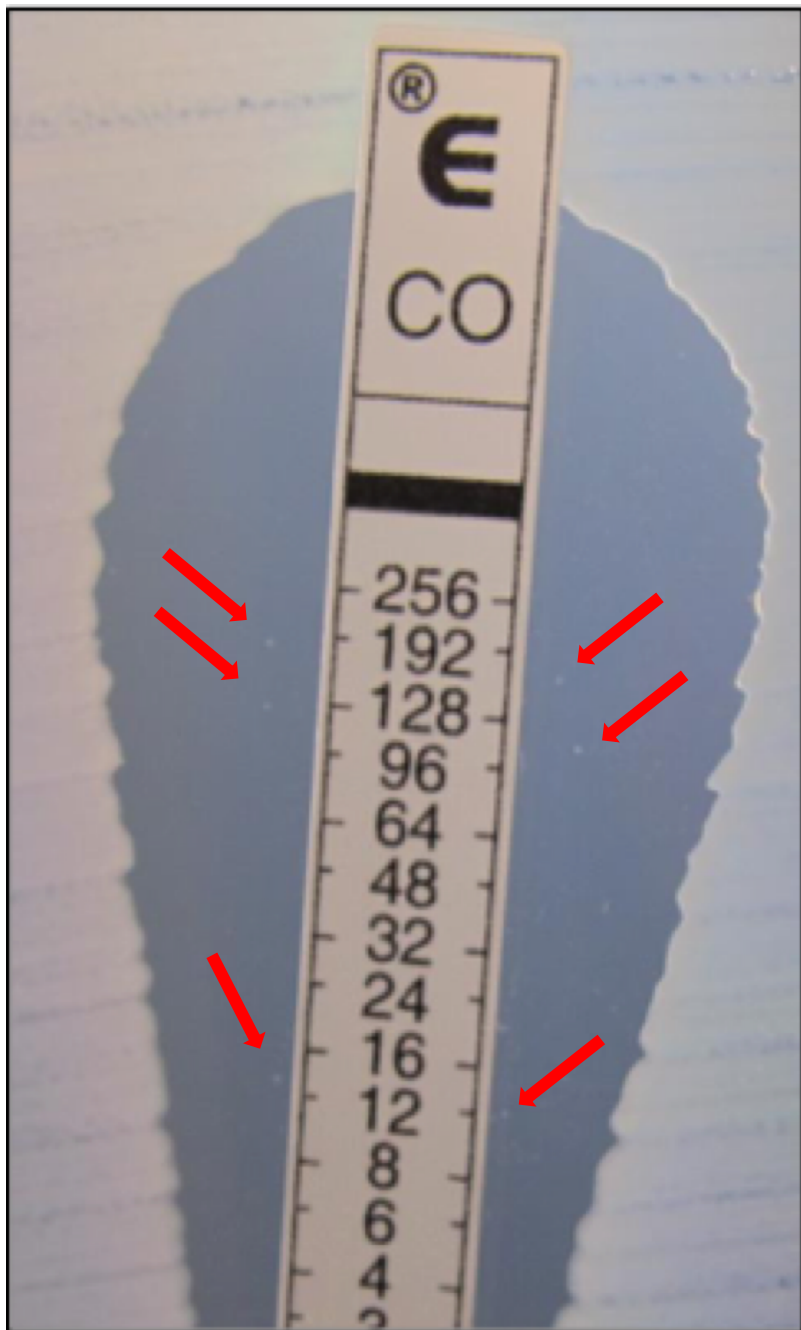

Supplement: Supplementary file 2 — Additional file 2: Supplementary Figure 2. Magnified image of E test's micro-colonies. Red arrows indicate resistant colonies. [file 12879_2022_7730_MOESM2_ESM.pdf]
