# Supplementary material for: Chromosomal coharboring of blaIMP-60 and mcr-9 in Enterobacter asburiae isolated from a Japanese woman with empyema: a case report
Source: BMC Infect Dis. 2022 Sep 30;22:762. doi: 10.1186/s12879-022-07730-7 (PMC9523918; doi:10.1186/s12879-022-07730-7)
Supplement: Supplementary file 3 — Additional file 3: Supplementary Table 1. Case Reports of Enterobacteriaceae carrying chromosomally located mcr-9 isolated from humans. [file 12879_2022_7730_MOESM3_ESM.docx]

**Supplementary material**

**Supplementary Table 1.** Case Reports of Enterobacteriaceae carrying chromosomally located *mcr-9* isolated from humans

| **Case** | **Year of onset** | **Age** | **Sex** | **Country** | **Pathogen** | **Susceptibility of Colistin** | **Method of colistin drug sensitivity test** | **Coharboring gene for antibiotics resistance** | **Treatment** | **Prognosis** | **Reference** | **Reference No.** |
| --- | --- | --- | --- | --- | --- | --- | --- | --- | --- | --- | --- | --- |
| 1 | 2018 | N/A | N/A | Japan | *Enterobacter asburiae* | S (0.125) | Broth microdilution and E-test | *bla_ACT-6_*, *bla_IMP-1_*, *mcr-9*, *sul1* | N/A | N/A | Pegah Kananizadeh, et al. BMC Infect Dis 2020.20:282:1-8 | 10 |
| 2 | 2010 | 86 | female | Israel | *Citrobacter telavivum* | S (0.5) | Broth microdilution or agar dilution | *bla_TEL-1_*, *mcr-9* | N/A | N/A (colonization) | Teresa Goncalves Ribeiro, et al. Eur J Clin Microbiol Infect Dis. 2021;40:123-31 | 11 |
| 3 | 2019 | 56 | male | Italy | *Enterobacter kobei* | S (8) | Broth microdilution | *fosA*, *mcr-9*, *bla_ACT-9_* | N/A | N/A (colonization for rectal swap) | Vittoria Mattioni Marchetti, et al. Diagnostics. 2021;11:79 | 12 |
|  |  |  |  |  |  |  |  |  |  |  |  |  |
|  |  |  |  |  |  |  |  |  |  |  |  |  |
|  |  |  |  |  |  |  |  |  |  |  |  |  |
